# FLASH-induced DNA damage reduction measured *in vitro* correlates with effective oxygen depletion determined *in silico*: further support for oxygen depletion contributing to FLASH’s reduced damage burden *in vitro*

**DOI:** 10.1093/bjr/tqaf097

**Published:** 2025-05-06

**Authors:** Bethany Rothwell, Christian R Cooper, Donald J L Jones, Michael J Merchant, Norman F Kirkby, Karen J Kirkby, Kristoffer Petersson, Jan Schuemann, George D D Jones

**Affiliations:** Physics Division, Department of Radiation Oncology, Massachusetts General Hospital & Harvard Medical School, Boston, MA 02114, United States; Oxford Institute for Radiation Oncology, Department of Oncology, University of Oxford, Oxford, OX3 7DQ, United Kingdom; Leicester Cancer Research Centre, Department of Genetics, Genomics & Cancer Sciences, University of Leicester, Leicester, LE1 7RH, United Kingdom; Leicester Cancer Research Centre, Department of Genetics, Genomics & Cancer Sciences, University of Leicester, Leicester, LE1 7RH, United Kingdom; Division of Cancer Sciences, Faculty of Biology, Medicine and Health, The University of Manchester, Manchester, M13 9PL, United Kingdom; Division of Cancer Sciences, Faculty of Biology, Medicine and Health, The University of Manchester, Manchester, M13 9PL, United Kingdom; Division of Cancer Sciences, Faculty of Biology, Medicine and Health, The University of Manchester, Manchester, M13 9PL, United Kingdom; Oxford Institute for Radiation Oncology, Department of Oncology, University of Oxford, Oxford, OX3 7DQ, United Kingdom; Radiation Physics, Department of Haematology, Oncology and Radiation Physics, Skåne University Hospital, Lund, 221 85, Sweden; Physics Division, Department of Radiation Oncology, Massachusetts General Hospital & Harvard Medical School, Boston, MA 02114, United States; Leicester Cancer Research Centre, Department of Genetics, Genomics & Cancer Sciences, University of Leicester, Leicester, LE1 7RH, United Kingdom

**Keywords:** FLASH radiotherapy, transient oxygen depletion, damage burden

## Abstract

**Objectives:**

FLASH irradiation demonstrates notable normal-tissue protective effects, including reduced damage *in vitro*. Radiochemical mechanisms proposed include radical-radical recombination and transient oxygen depletion (TOD), but the relative contributions remain unclear. This study compares FLASH-mediated DNA damage reduction *in vitro* with oxygen depletion for FLASH radiotherapy modelled *in silico*, to (i) investigate the contribution of TOD towards the reduced damage burden *in vitro*, and (ii) evaluate its contribution to the broader FLASH effect *in vivo*.

**Methods:**

An *in silico* model was used to identify and compare the parameter space for FLASH-induced oxygen depletion in an *in-vitro* setup with experimental DNA damage reduction data, previously determined using the alkaline comet assay *ex vivo*.

**Results:**

Correlation analysis revealed a strong relationship between model-predicted oxygen depletion and experimentally-observed DNA damage reduction (Spearman’s = 0.87, *P* = 2 × 10^−6^; Pearson’s = 0.85, *P* = 4 × 10^−6^).

**Conclusions:**

Findings support a significant role for TOD in the FLASH-induced reduction in damage *in vitro* at low oxygen tensions. However, parameter spaces identified, for both oxygen depletion *in silico* and DNA damage reduction *in vitro*, suggest that TOD may only partially contribute to the wider-ranging FLASH sparing effects *in vivo*. Further work is required to clarify this.

**Advances in knowledge:**

Findings support TOD as a key mechanism for the reduced damage burden of FLASH *in vitro*. However, further work is required to demarcate the sparing effects of FLASH *in vivo*.

## Introduction

Numerous studies have demonstrated the normal tissue-sparing effects of higher dose rate “FLASH” irradiation *in vivo*, with a reduction in damage burden also reported *in vitro*.^1–3^ Towards the latter, two principal radiochemical mechanisms have been proposed: radical-radical recombination (RRR) and transient oxygen depletion (TOD),^2^^,^^4^ both being proposed to lead to lower levels of FLASH-induced damage.

Recently, Cooper et al^5^ reported that FLASH irradiation at low oxygen tension induces lower levels of DNA damage in whole-blood peripheral blood lymphocytes (PBLs) irradiated *ex vivo*, an effect modulated by oxygen tension, dose, and dose rate. This supports the concept of an oxygen-related mechanism contributing to the damage-sparing effect of FLASH irradiation *in vitro*,^2^ but this study failed to distinguish between RRR and TOD as being the mechanism(s) responsible. However, further comet assay analysis was undertaken to assess crosslink formation as a putative marker of RRR (particularly, if any organic radicals recombine) and also to assess anoxic DNA damage formation as an indicative marker of TOD.^6^ The findings of this study were that, following FLASH irradiation, there was no evidence of any crosslink formation, so no experimental evidence of RRR; however, FLASH irradiation induced a more anoxic profile of induced damage, supporting the TOD mechanism as being a key driver of the reduced damage burden witnessed *in vitro*.

In parallel studies, Rothwell et al^7^ reported an *in silico* model to determine the parameters for oxygen depletion from FLASH radiotherapy. This used an eight-dimensional parameter space to demonstrate conditions under which radiation may induce effective depletion of oxygen, sufficient to enable a diffusion-limited hypoxic cellular response. Findings suggest that FLASH sparing by oxygen depletion is best achieved using higher doses, delivered at dose rates of tens of Gy/s or higher, but only for systems of limited oxygen tension at the time of irradiation.

Building on our aforementioned previous studies, we presently investigate the extent to which oxygen depletion may contribute to FLASH-induced reduction in damage *in vitro*, by comparing the parameter space for the experimentally determined FLASH DNA damage sparing of Cooper et al^5^ with the parameter space for the depletion of oxygen for FLASH irradiation determined *in silico,* simulating the experimental *in vitro* setup.^7^ By integrating computational modelling with experimental findings, this approach provides a more comprehensive understanding of the parameter dependencies involved. We then assess the extent to which TOD may contribute to the full “FLASH effect” *in vivo*.

## Methods

To identify the parameter space for FLASH-induced oxygen depletion for the specific combinations of dose, dose rate, and oxygen tension tested by Cooper et al,^5^ simulations were generated using the oxygen depletion model described in Rothwell et al^7^ using relevant biological parameters to match the *in-vitro* experimental conditions studied. Radiolytic depletion was calculated using a 2-stage model in which generic radiolytic species are generated as a function of dose (at a rate of $3\times 10^{-4}$ mol/(m^3^.Gy)^8^^,^^9^) and react with available O_2_, as described previously.^7^ However, it should be noted that reported values of oxygen consumption rate vary significantly in the literature, and simulated oxygen depletion predictions are highly sensitive to this parameter—see Supplementary Materials for further details.

Changes in radiosensitivity were characterized using the oxygen enhancement ratio (OER), calculated as a function of oxygen concentration.^7^ The FLASH sparing metric is defined as the ratio of the OER value at the depleted oxygen level (the point at which the full dose has been applied) to the OER value under conditions with no net oxygen depletion, this being termed the “TOD ratio.” Parallel coordinate plots were generated to show the effect of multiple parameters on the determined TOD ratios.^7^

To identify the parameter space for the FLASH-mediated reduction in DNA damage formation reported by Cooper et al,^5^ the model was adapted to assess the DNA damage ratios obtained for the experimental data. Here, the experimental FLASH sparing metric was defined as the ratio of damage for each parameter combination at higher dose rates (>0.1 Gy/s), relative to the corresponding conventional (CONV) dose rate (0.1 Gy/s), determined by mean values of %Tail DNA as $\frac{\mathrm{FLASH}-\mathrm{control}}{\mathrm{CONV}-\mathrm{control}}$, this being termed the “damage ratio”; % Tail DNA is regarded as the most robust/dynamic comet assay measure of DNA damage, calculated as the percentage of total comet DNA present in the comet tail for 50 assessed/scored comets.^5^ Again, parallel coordinate plots were generated to show the impact of the parameter combinations on the damage ratios.

Finally, the respective TOD and damage ratios determined for each combination of the conditions investigated were correlated to determine whether predicted TOD corresponds to lower levels of DNA damage for FLASH versus CONV irradiation *in vitro*.^5^

## Results

**Figure 1.**
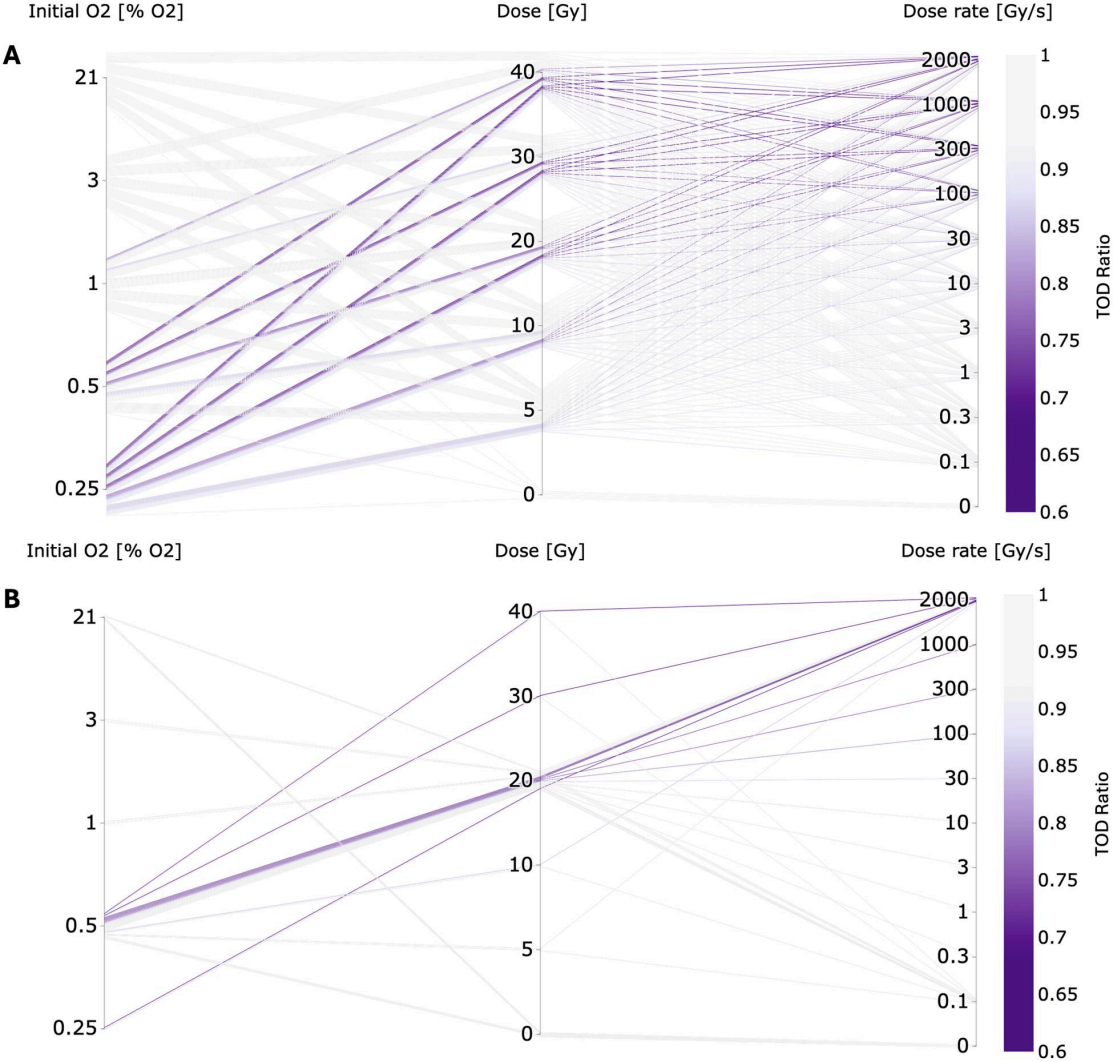
(A) Parallel coordinates plot of the *in silico*-generated TOD ratios for all possible combinations of the 5 initial oxygen concentrations, 5 non-zero total doses, and 10 dose rates reported by Cooper et al,^5^ generating 250 possible combinations (255 including five 0 Gy measurements at each initial oxygen value). (B) Parallel coordinates plot of the TOD ratios for the 36 combinations of initial oxygen concentration, total dose, and dose rate studied by Cooper et al,^5^ as denoted in Table 1. The greater intensity of purple of each respective parameter line represents a greater increase in radioresistance from TOD. Abbreviation: TOD = transient oxygen depletion.

Figure 1 depicts simulation data from the model determined TOD ratios. This is shown in Figure 1A for all 250 possible combinations of the parameters reported in the experimental study (5 initial oxygen concentrations, 5 non-zero total doses, and 10 dose rates), and in Figure 1B for the 36 parameter combinations actually investigated by the comet assay in the experimental study,^5^ as denoted in Table 1. A maximum ratio of 1 indicates no change in OER from radiation-induced oxygen depletion. Values close to 1 are indicative of the CONV (0.1 Gy/s) case, where net oxygen depletion is negligible due to low depletion (for low total doses) or efficient oxygen recovery over the time of irradiation (for low dose rates). The greater intensity of each respective parameter line represents a lower TOD ratio and therefore a greater predicted increase in oxygen depletion and radioresistance. Consequently, this demonstrates greater FLASH sparing being associated with decreased oxygen tension, together with increased dose rate and total dose delivered.

**Table 1.** Parameter values used in the Cooper et al (2022), comet assay study and in the current simulation.

Parameter	Values used	No. of comet measurements
Initial oxygen level (% O_2_)	0.25	2 controls (0 Gy)
	0.5	5 FLASH (2000 Gy/s)
	1	5 CONV (0.1 Gy/s)
	3	
	21	(12 measurements)
Dose (Gy)	5	2 controls (0 Gy)
	10	5 FLASH (2000 Gy/s)
	20	5 CONV (0.1 Gy/s)
	30	
	40	(12 measurements)
Dose rate (Gy/s)	0.1	2 controls (0 Gy)
	0.3	1 CONV (0.1 Gy/s)
	1	9 “non-CONV” (>0.1 Gy/s)
	3	
	10	(12 measurements)
	30	
	100	
	300	
	1000	
	2000	

Abbreviation: CONV = conventional.

**Figure 2.**
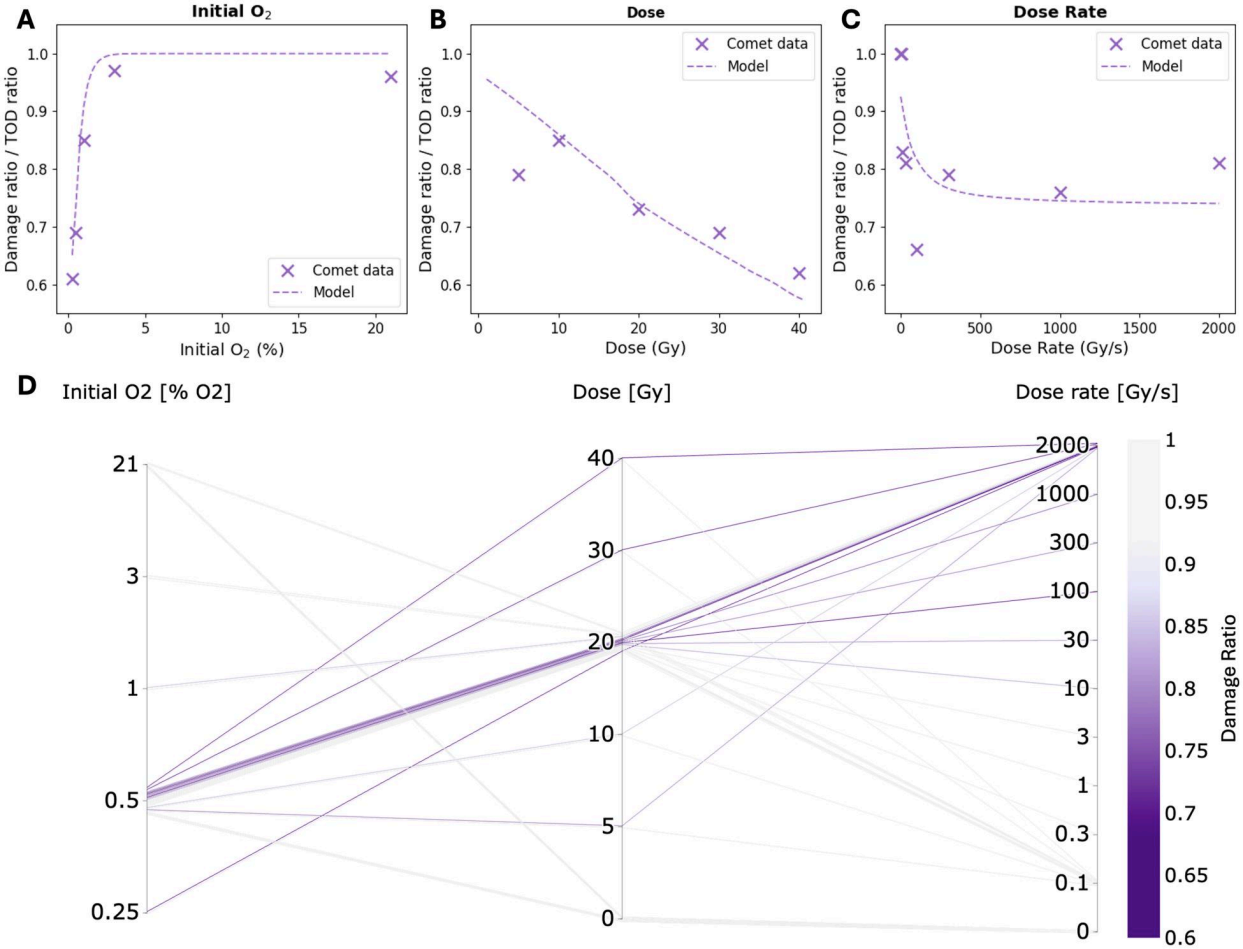
Plots showing the DNA damage ratios reported from the experimental datasets of Cooper et al^5^ for (A) initial oxygen level, (B) total dose delivered, and (C) dose rate of the delivery. The simulated TOD ratios calculated using the oxygen model under equivalent experimental conditions are also shown for each parameter (dashed line) and compared to the experimental damage ratios (x-points). (D) A parallel coordinates plot of the damage ratio obtained for each combination of these parameters depicted for these 3 datasets. The greater intensity of purple for each respective parameter line represents less relative damage from higher dose rates compared to CONV radiation.

Figure 2A-C show the determined damage ratios for the 3 reported experimental datasets of Cooper et al^5^ for variations in initial oxygen level, total dose delivered, and dose rate of delivery, respectively (see Table 1). The TOD ratio results from the oxygen model under the same experimental conditions are also shown for each parameter. Figure 2D depicts the merger of these 3 experimental datasets, showing the 36 DNA damage ratios calculated from the experimental data.^5^ A greater intensity for each respective parameter line represents less relative damage from FLASH compared to CONV irradiation. Similar to Figure 1B, Figure 2D shows a greater FLASH damage sparing being associated with a decrease in oxygen tension (≲0.5% O_2_), and an increase in dose rate (≳30 Gy/s) and total dose (≳20 Gy).

**Figure 3.**
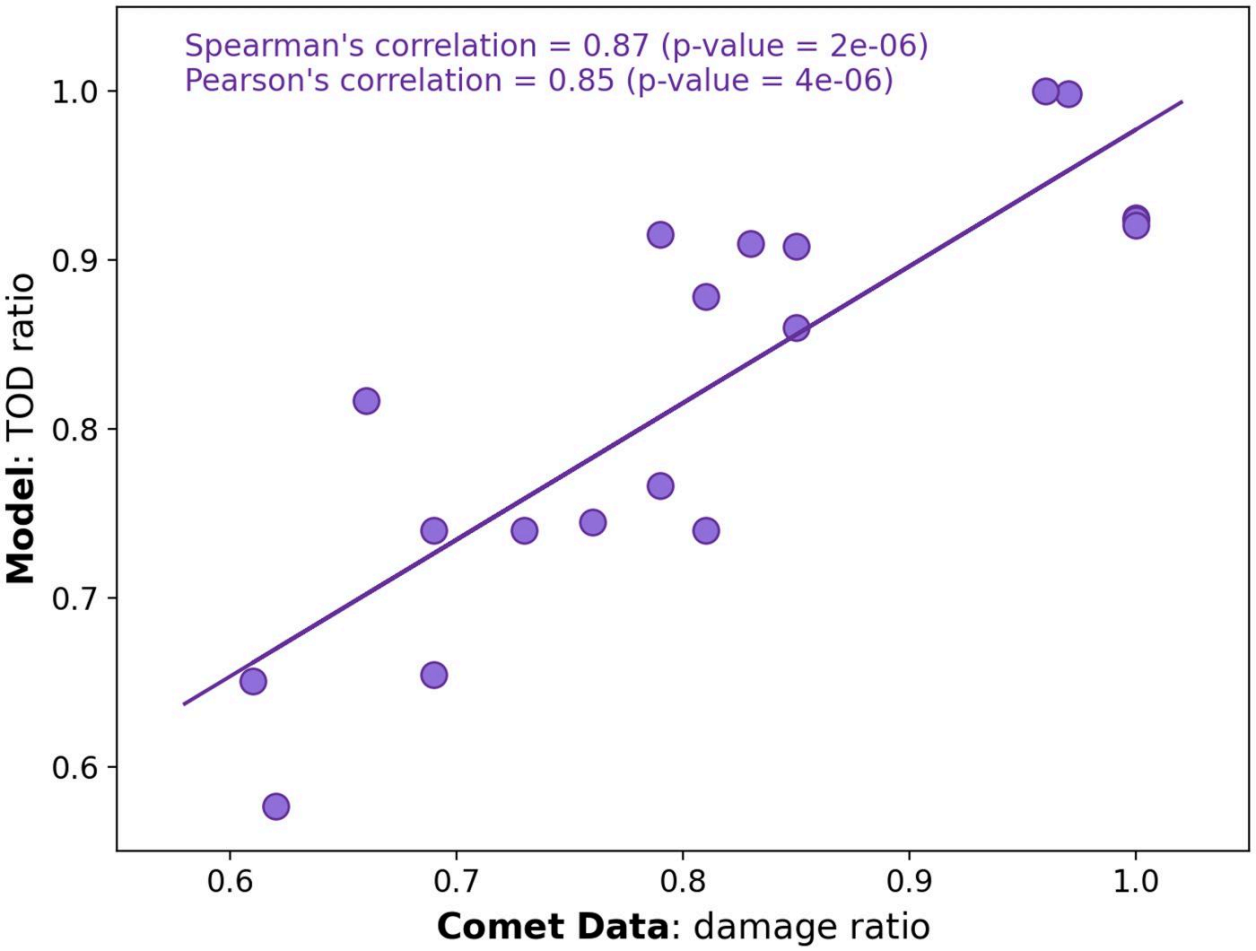
Direct correlation of the 19 paired combinations of initial oxygen concentration, total dose, and dose rate used to calculate the DNA damage ratios using the experimental data of Cooper et al^5^ versus the modelled TOD ratios for the same 19 combinations. Values close to 1 are indicative of both negligible oxygen depletion and low damage reduction, while values progressively <1 reflect greater oxygen depletion correlating lower induced damage levels. Abbreviation: TOD = transient oxygen depletion.

Of the 36 experimental parameter combinations studied,^5^ 19 were used for a correlation study, disregarding 6 zero-dose control measurements, and 11 measurements at 0.1 Gy/s used as the reference for FLASH versus CONV ratio calculations; these exclusions prevent a skewing of the analysis by an over-representation of fixed-point control data in determining the ratio calculations. Figure 3 depicts the correlation of the damage ratios determined for the 19 combinations of initial oxygen concentration, total dose and dose rate selected versus the modelled TOD ratios for the same 19 combinations. The determined Spearman’s correlation was 0.87 and highly significant (*P* = 2 × 10^−6^), as was the Pearson’s correlation (0.85; *P* = 4 × 10^−6^).

## Discussion

To better understand the extent to which TOD may contribute to the FLASH-induced reduction in damage burden witnessed *in vitro*, the present study compared the comet assay data of Cooper et al^5^ against an *in silico* model for oxygen depletion.^7^ The precedent for this comparison arises from the proposal that the shorter timescale of FLASH exposure leads to a higher concentration of radiation-induced secondary and tertiary organic radicals, which transiently consumes local oxygen. This in turn may lead to lower radiochemical yields of immediate strand break damage witnessed under anoxia,^10^ together with the greater thiol-mediated chemical “repair” of radiation-induced secondary and tertiary organic radicals. Both mechanisms ultimately lead to lower levels of radiation-induced damage formation following FLASH irradiation, the latter being the well-established oxygen fixation versus chemical-repair hypothesis for radiation-induced cellular damage manifestation.^11^ The alkaline comet assay detects strand break damage resulting from both single and double strand breaks, plus strand breaks resulting from alkali labile sites (non-strand broken lesions that form strand breaks under alkali conditions).^12^ So, in effect, the alkaline comet assay is a “catch-all” for all cellular strand break damage plus certain further damage, making it is an ideal protocol with which to assess the effects of FLASH on damage burden.

Correlation of the modelled TOD ratios versus the experimentally obtained DNA damage ratios revealed significant correlations (Spearman’s correlation of 0.87; *P* = 2 × 10^−6^, and Pearson’s correlation of 0.85; *P* = 4 × 10^−6^) and the same observed behaviour; notably that reductions in damage and OER were best observed for sufficiently low initial oxygen levels, sufficiently high doses, and sufficiently high dose rates. This is indicative of greater changes in OER/oxygen depletion correlating with lower levels of DNA damage for FLASH versus CONV. While this correlation does not establish absolute causation, it lends further support to oxygen depletion as the key mechanism underpinning the FLASH-induced reduction in damage burden witnessed *in vitro*.

However, this may not account for the full FLASH effect *in vivo*. The rate of radiochemical oxygen consumption (3×10^−4 ^mol/(m^3^.Gy)) used in this modelling study, supported by oxygen depletion measurements,^8^^,^^9^ can account for significant OER shifts using relevant doses (≳20 Gy), but only under conditions of low initial oxygen tension (≲0.5% O_2_). This rate aligns with real-time oxygen measurements in mouse skin during FLASH irradiation,^9^ where similar setups have shown a FLASH effect of 15%-50%,^3^^,^^13^ For example, Zhang et al^3^ used a dose rate of 130 Gy/s and dose of 27 Gy to observe a ∼25% reduction in skin contraction. However, modelling these parameters with an initial oxygen level of 20-40 mmHg, as measured by Cao et al,^9^ predicts a maximum OER change of less than 0.5%. Even with a dose rate of 2000 Gy/s, as used by Cooper et al,^5^ a dose exceeding 250 Gy would be required for a similar sparing effect.

However, there is considerable variability in reported radiochemical consumption rates, ranging from ∼1×10^−4^ measured in water,^8^ up to ∼8×10^−4 ^mol(m^3^.Gy) measured intracellularly in mice,^14^ and up to ∼3×10^−3^ measured in chemical systems with various reducing agents.^15^ There is also variation across different radiation modalities, beam characteristics, and experimental setups (see Supplementary Materials). As shown in Figure S1, the predicted oxygen-depletion-induced sparing effect is highly sensitive to this parameter which, even in the range of literature-derived values, can ultimately determine whether the effect occurs. For higher values of oxygen consumption rate, TOD may become feasible *in vivo*, depending on other parameters.^7^ Therefore, accurately determining the radiolytic oxygen rate is crucial, as small variations could significantly alter the feasibility of TOD for FLASH.

Nevertheless, achieving a ∼25% sparing effect while maintaining the dose rate used by Zhang et al^3^ would require an oxygen consumption rate of about $4\times 10^{-3}$mol/(m^3^.Gy) (which exceeds current reported values) or a significantly lower initial oxygen concentration of around 5 mmHg. The latter scenario may be plausible; studies have shown skin oxygen tension in anaesthetized rats as low as 5-10 mmHg.^16^ Furthermore, oxygen heterogeneity, particularly between capillaries,^17^ can be extreme, potentially giving rise to near-hypoxic niches^18^ which could undergo significant changes in OER-dependent radiosensitivity that impact the whole system. Experimental oxygen measurements typically represent an average,^9^ yet oxygen partial pressures vary substantially across different regions of human tissue. For example, arterial blood oxygen tensions typically range from 90-100 mmHg, dropping to around 30-40 mmHg in venous blood and 20-40 mmHg in capillaries.^7^ In the extracellular matrix, oxygen tension is generally lower, around 10-20 mmHg, and can drop as low as 1-2 mmHg in hypoxic regions within tissues such as the skin and brain—these spatial variations are difficult to measure accurately.^19^

Finally, this study also highlights how experimental and computational approaches can be combined in elucidating the mechanism for FLASH. While experimental work serves to support computational models (and vice versa), simulations can be used to generate significantly more data points. Here, 36 measurements were made experimentally, with the majority at 0.5% O_2_, using 20 Gy at either 2000 or 0.1 Gy/s (as these were the default values for each parameter while a single separate parameter was varied), while 250 measurements were generated by the model using every parameter combination. Complementing experimental findings with computational predictions can provide a more comprehensive understanding of the parameter dependencies involved (eg, Figure 1A) and address any “data gaps” within experimental work where measurements are not feasible.

In conclusion, our findings support TOD as a key mechanism responsible for the reduced damage burden of FLASH exposure witnessed *in vitro* at low oxygen tension. Furthermore, we highlight the need for better determinations of radiogenic oxygen consumption rates and heterogeneous oxygen distributions *in vivo*; these factors and others will be incorporated and further evaluated in ongoing work focusing on FLASH-induced oxygen depletion *in vivo*.

## Supplementary Material

tqaf097_Supplementary_Data
