# Supplementary material for: FLASH-induced DNA damage reduction measured in vitro correlates with effective oxygen depletion determined in silico: further support for oxygen depletion contributing to FLASH’s reduced damage burden in vitro
Source: Br J Radiol. 2025 May 6;98(1171):1032–7. doi: 10.1093/bjr/tqaf097 (PMC12202011; doi:10.1093/bjr/tqaf097)
Supplement: tqaf097_Supplementary_Data [file tqaf097_supplementary_data.zip › tqaf097_Supplementary_Data/Supplementary_materials_noFigure_doubleSpaced.docx]

**Supplementary Materials**

Sensitivity of TOD Predictions to Oxygen Consumption Rates

There is substantial variation in reported oxygen consumption rates in the literature due to differences in experimental systems, radiation modalities, and measurement conditions. Table S1 presents examples from studies using photon, proton and carbon irradiation^1–5^. Here, data from in-solution measurements conducted in closed chemical systems are included. While *in vivo* measurements of oxygen depletion have been reported^2,4,6^, this analysis focuses on the impact of changes in this single model parameter rather than the net effect of oxygen diffusion and consumption measured *in vivo*. As shown, reported values span an order of magnitude, from approximately 1 × 10^-4^ to 3 × 10^-3^ mol/(m^3^.Gy). In particular, Koch et al. demonstrated that the measured oxygen consumption rate is highly dependent on experimental conditions, including the choice of reducing agents, buffer composition, and dose rate. Their findings highlight the sensitivity of oxygen consumption to differences in the cellular environment and beam modality.

To assess how variability in oxygen consumption rates influences model predictions, we performed a sensitivity analysis evaluating the impact of different reported values on the transient oxygen depletion (TOD) ratio for FLASH and conventional (CONV) irradiation predicted by the model^7^. Figure S1 presents TOD ratios across the range of oxygen consumption rates reported in the literature, simulated using the parameters in this study: 20 Gy dose, initial oxygen concentration of 0.5% O_2_, and dose rates of 2000 Gy/s (FLASH) and 0.1 Gy/s (CONV).

In this study, we used an oxygen consumption rate of 3 × 10^-4^ mol/(m^3^·Gy), which aligns well with experimental comet assay data^8^ and provides a conservative estimate for oxygen-depletion-induced FLASH sparing. However, as shown in Figure S1, the predicted oxygen-depletion-induced sparing effect is highly sensitive to this parameter. A maximal effect (TOD ≈ 0.5) is observed for oxygen consumption rates ≥ 8 × 10^-4^ mol/(m^3^·Gy), while the effect diminishes sharply for rates < 2 × 10^-4^ mol/(m^3^·Gy). If the oxygen consumption rate is much lower than the assumed value, the model predicts minimal or no FLASH effect, whereas higher values could lead to a sparing effect exceeding experimentally observed levels. However, while this parameter strongly influences the magnitude of TOD, the overall trends and correlations with experimental data outlined in this study remain consistent.

It should be noted that several studies have shown that oxygen consumption rate decreases as dose rate increases, a factor we aim to incorporate into future iterations of the model. However, as shown in Table S1, the reported values remain highly variable across different studies and radiation modalities, making it difficult to derive a universally applicable dose-rate-dependent formula from existing literature data.

**Table S1**: Examples of literature-reported measurements of radiolytic oxygen consumption in sealed chemical systems.

| **Study** | **System** | **Radiation type** | **Dose rate** | **O_2_ consumption (x10^-4^ mol/(m^3^.Gy))** |
| --- | --- | --- | --- | --- |
| Jansen et al. 2021 | Pure water | 224 kV photons | 1 | 1.75 |
|  |  |  | 2.4 | 0.94 |
|  |  |  | 8.1 | 1.09 |
|  |  |  | 52 | 0.62 |
|  |  | 224 MeV protons | 2 | 3.1 |
|  |  |  | 5 | 2.3 |
|  |  |  | 20 | 1.8 |
|  |  | 400 MeV/u carbon | 2.55 | 1.3 |
|  |  |  | 5 | 1.6 |
| Cao et al. 2021 | 5% BSA | 10 MeV electrons | 0.1 | 3.4-3.7 |
|  |  |  | 300 | 2.8-3.0 |
| El Khatib et al. 2022 | 5% BSA | 230 MeV protons | 0.6 | 5.5 |
|  |  |  | 100 | 4.9 |
| Van Slyke et al. 2022 | "CELL" solution | 230 MeV protons | 0.5 | 9.6 |
|  |  |  | 20 | 8 |
|  |  |  | 100 | 7.1 |
| Koch et al 2023 | Various reducing agents | Cs irradiator | 0.1 | $\sim$1-30 |

**Figure S1**: Simulated transient oxygen depletion (TOD) ratio as a function of oxygen consumption rate. TOD ratios were modelled for FLASH (2000 Gy/s) and conventional (0.1 Gy/s) irradiation across a range of oxygen consumption rates reported in the literature. Simulations used a 20 Gy dose and an initial oxygen concentration of 0.5% O_2_. In the study, an oxygen consumption rate of 3 × 10^-4^ mol/(m^3^·Gy) was used and compared to the experimental comet assay data.

**References:**

1. Jansen J, Knoll J, Beyreuther E, et al. Does FLASH deplete oxygen? Experimental evaluation for photons, protons, and carbon ions. *Med Phys*. 2021;48(7):3982-3990. doi:10.1002/mp.14917

2. Van Slyke AL, El Khatib M, Velalopoulou A, et al. Oxygen Monitoring In Vitro and In Vivo During Proton Irradiation at Conventional and FLASH Dose Rates. *Radiat Res*. 2022;198(2):181-189. doi:10.1667/RADE-21-00232.1

3. Khatib M El, Van Slyke AL, Velalopoulou A, et al. Ultrafast Tracking of Oxygen Dynamics during Proton FLASH. *International Journal of Radiation Oncology*Biology*Physics*. 2022;113(3):624-634. doi:10.1016/j.ijrobp.2022.03.016

4. Cao X, Zhang R, Esipova T V, et al. Quantification of oxygen depletion during FLASH irradiation in vitro and in vivo. *Int J Radiat Oncol Biol Phys*. Published online 2021:1-9. doi:10.1016/j.ijrobp.2021.03.056

5. Koch C, Kim M, Wiersma R. Radiation-Chemical Oxygen Depletion Depends on Chemical Environment and Dose-Rate: Implications for the ‘FLASH’ Effect. *International Journal of Radiation Oncology*Biology*Physics*. Published online April 2023. doi:10.1016/j.ijrobp.2023.04.001

6. Petusseau AF, Clark M, Bruza P, Gladstone D, Pogue BW. Intracellular Oxygen Transient Quantification in Vivo During Ultra-High Dose Rate FLASH Radiation Therapy. *Int J Radiat Oncol Biol Phys*. Published online 2024. doi:10.1016/j.ijrobp.2024.04.068

7. Rothwell BC, Kirkby NF, Merchant MJ, et al. Determining the parameter space for effective oxygen depletion for FLASH radiation therapy. *Phys Med Biol*. 2021;66:055020. doi:10.1088/1361-6560/abe2ea

8. Cooper CR, Jones D, Jones GD, Petersson K. FLASH irradiation induces lower levels of DNA damage ex vivo, an effect modulated by oxygen tension, dose, and dose rate. *British Journal of Radiology*. 2022;95(1133). doi:10.1259/bjr.20211150
