# Supplementary figures and images for: FLASH-induced DNA damage reduction measured in vitro correlates with effective oxygen depletion determined in silico: further support for oxygen depletion contributing to FLASH’s reduced damage burden in vitro
Source: Br J Radiol. 2025 May 6;98(1171):1032–7. doi: 10.1093/bjr/tqaf097 (PMC12202011; doi:10.1093/bjr/tqaf097)

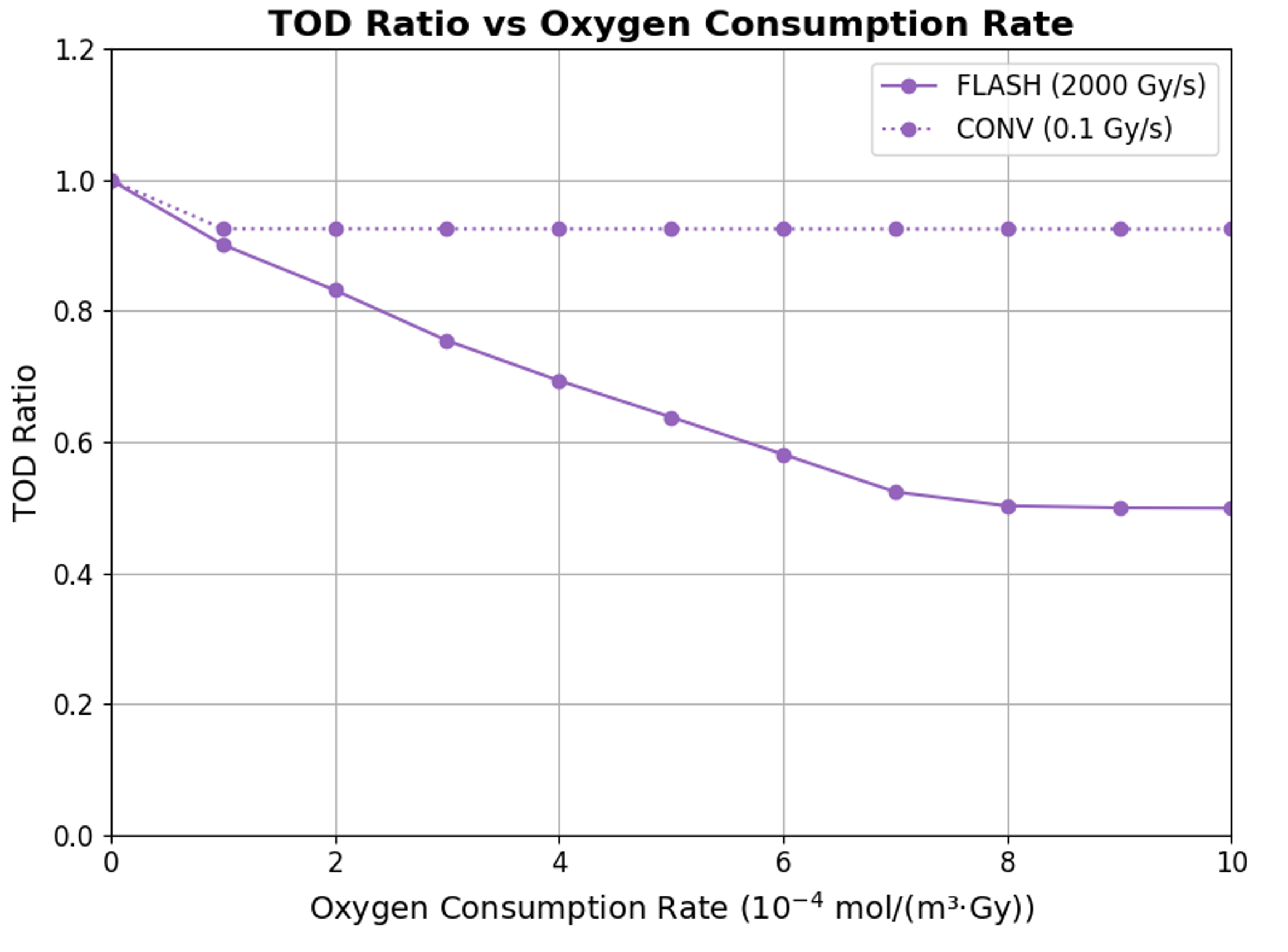

Supplement: tqaf097_Supplementary_Data [file tqaf097_supplementary_data.zip › tqaf097_Supplementary_Data/Supplementary_Figure_S1.tiff]
